# “Tricking the Brain” Using Immersive Virtual Reality: Modifying the Self-Perception Over Embodied Avatar Influences Motor Cortical Excitability and Action Initiation

**DOI:** 10.3389/fnhum.2021.787487

**Published:** 2022-02-09

**Authors:** Karin A. Buetler, Joaquin Penalver-Andres, Özhan Özen, Luca Ferriroli, René M. Müri, Dario Cazzoli, Laura Marchal-Crespo

**Affiliations:** ^1^Motor Learning and Neurorehabilitation Laboratory, ARTORG Center for Biomedical Engineering Research, University of Bern, Bern, Switzerland; ^2^Psychosomatic Medicine, Department of Neurology, University Hospital of Bern (Inselspital), Bern, Switzerland; ^3^Gerontechnology and Rehabilitation Group, ARTORG Center for Biomedical Engineering Research, University of Bern, Bern, Switzerland; ^4^Department of Neurology, University Neurorehabilitation, University Hospital of Bern (Inselspital), University of Bern, Bern, Switzerland; ^5^Neurocenter, Luzerner Kantonsspital, Lucerne, Switzerland; ^6^Department of Cognitive Robotics, Delft University of Technology, Delft, Netherlands

**Keywords:** embodiment, body illusion, self-perception, motor cortex, motor evoked potentials (MEPs), motor control, immersive virtual reality (IVR)

## Abstract

To offer engaging neurorehabilitation training to neurologic patients, motor tasks are often visualized in virtual reality (VR). Recently introduced head-mounted displays (HMDs) allow to realistically mimic the body of the user from a first-person perspective (i.e., avatar) in a highly immersive VR environment. In this immersive environment, users may embody avatars with different body characteristics. Importantly, body characteristics impact how people perform actions. Therefore, alternating body perceptions using immersive VR may be a powerful tool to promote motor activity in neurologic patients. However, the ability of the brain to adapt motor commands based on a perceived modified reality has not yet been fully explored. To fill this gap, we “tricked the brain” using immersive VR and investigated if multisensory feedback modulating the physical properties of an embodied avatar influences motor brain networks and control. Ten healthy participants were immersed in a virtual environment using an HMD, where they saw an avatar from first-person perspective. We slowly transformed the surface of the avatar (i.e., the “skin material”) from human to stone. We enforced this visual change by repetitively touching the real arm of the participant and the arm of the avatar with a (virtual) hammer, while progressively replacing the sound of the hammer against skin with stone hitting sound via loudspeaker. We applied single-pulse transcranial magnetic simulation (TMS) to evaluate changes in motor cortical excitability associated with the illusion. Further, to investigate if the “stone illusion” affected motor control, participants performed a reaching task with the human and stone avatar. Questionnaires assessed the subjectively reported strength of embodiment and illusion. Our results show that participants experienced the “stone arm illusion.” Particularly, they rated their arm as heavier, colder, stiffer, and more insensitive when immersed with the stone than human avatar, without the illusion affecting their experienced feeling of body ownership. Further, the reported illusion strength was associated with enhanced motor cortical excitability and faster movement initiations, indicating that participants may have physically mirrored and compensated for the embodied body characteristics of the stone avatar. Together, immersive VR has the potential to influence motor brain networks by subtly modifying the perception of reality, opening new perspectives for the motor recovery of patients.

## Introduction

Stroke represents a leading cause of long-term disability in adults worldwide, with one-third of chronic stroke patients requiring assistance during activities of daily living (Feigin et al., 2014). Intensive and costly neurorehabilitation interventions are an integral part of the therapy following stroke, aiming at regaining (part of) the motor functionality of patients. Within this context, robotic neurorehabilitation has been receiving increasing interest to provide more cost-effective therapy (Lum et al., 2012). Robotic-assisted interventions allow for repetitive, high-intensity, and task-specific training, lowering costs and personal limitations (e.g., fatigue) and optimizing the potential of motor recovery of patients (Marchal-Crespo and Reinkensmeyer, 2009).

To increase the engagement of patients during training, motor tasks are often visualized in virtual reality (VR), allowing the simulation of various real and imaginary activities of daily living (Lee et al., 2003; Perez-Marcos et al., 2018). VR further offers the possibility to individualize the virtual environment to the needs of the patients, and to provide standardized and safe training (Rose et al., 2005; Marchal-Crespo and Reinkensmeyer, 2008). A large body of research has demonstrated the efficacy of VR therapy in (robotic) stroke rehabilitation (Adamovich et al., 2004; Deutsch et al., 2004; Jang et al., 2005). However, in standard clinical VR settings, computer screens are used to display the virtual training environment. Here, the patient interacts with the virtual elements using an abstract virtual representation (e.g., a cursor). While this symbolic interaction provides useful visual guidance, it strongly deviates from interactions required in the real world and, therefore, may limit the transfer of acquired skills into activities of daily living (de Mello Monteiro et al., 2014; Bezerra et al., 2018).

Recently emerging head-mounted displays (HMDs) provide highly immersive virtual training environments. In this immersive virtual environment, the user interacts with a virtual self-representation perceived from first-person perspective (i.e., an avatar), realistically mimicking the body of the user. Previous work has suggested that immersive virtual reality, compared with screens, may further promote motor training because they enhance embodiment over the avatar (Wenk et al., 2021) i.e., the body of the avatar is –at least partially– processed like the own (virtual) body (Kilteni et al., 2012a). In the immersive virtual training environment, the user may experience the feeling of body ownership over the avatar. Body ownership –one out of the three components of embodiment together with agency [i.e., the feeling of initiating and being in control of the own actions; (e.g., David et al., 2008; Braun et al., 2018)] and location [i.e., the experienced location of the body in space; (e.g., Blanke, 2012)]– is the cognition that a body and/or its parts belong to oneself (Blanke, 2012). Body ownership results from the integration and interpretation of multimodal sensory information in the brain, importantly, visual, somatosensory, and proprioceptive signals (Botvinick and Cohen, 1998; Maravita et al., 2003; Ehrsson, 2004). Neuroimaging studies have shown that body ownership relies on frontal premotor, somatosensory, temporoparietal junction, and insular brain regions (Botvinick and Cohen, 1998; Maravita et al., 2003; Ehrsson, 2004; Tsakiris, 2010).

Even though our body and its physical features and capabilities (e.g., the size of body parts and/or the color or material of the skin) usually do not change –and one could assume that the perception of the own body is stable–a vast amount of research has shown that bodily self-perceptions are continuously updated in the brain in response to sensory signals related to the body characteristics (de Vignemont, 2010; Serino and Haggard, 2010; Tsakiris, 2010, 2017; Longo and Haggard, 2011; Blanke et al., 2015). Consequently, multisensory feedback can be used to modulate the self-perception of the body, as for example, in the well-known “rubber hand illusion” paradigm, first introduced by Botvinick and Cohen (1998). Here, an experimenter simultaneously strokes the hidden real hand of a participant and a rubber hand placed in front of the participant. The simultaneously felt stroking on the real hand and the visual perception of the rubber hand being stroked has been shown to reliably induce the feeling of body ownership over the rubber hand in the participant. The rubber hand illusion has also been demonstrated by providing auditory instead of visual feedback. In the “marble hand illusion,” Senna et al. (2014) touched the (hidden) hand of the participant with a hammer that was coupled with stone-hitting sound. This led participants to experience their own hand to be more stone-like than in the control condition (e.g., they rated their own hand as stiffer, heavier, harder, unnatural, and less sensitive). Various variations of the rubber hand illusion paradigm have shown that body ownership can be experimentally induced in a part of a body or an entire body other than one’s own in healthy young (Ehrsson, 2004; Tsakiris and Haggard, 2005; Tsakiris et al., 2006; Lloyd, 2007; Haans et al., 2008; Kammers et al., 2009; van der Hoort et al., 2011; Kalckert and Ehrsson, 2012; Lopez et al., 2012; Pozeg et al., 2014; Crea et al., 2015; Flögel et al., 2016; Wen et al., 2016; Burin et al., 2017; Riemer et al., 2019; Matsumoto et al., 2020), elderly (Burin and Kawashima, 2021), and neurologic patients (Zeller et al., 2011; Lenggenhager et al., 2012; Burin et al., 2015; Wawrzyniak et al., 2018). The demonstrated flexibility of the brain is indeed crucial to preserve a stable body image while the perceptual characteristics of the body constantly vary in everyday life. For example, the skin color may change depending on light, and the size and shape of body parts are influenced by posture and distance. Therefore, to save resources, the brain is trained to accept deviations resulting from a mismatch between sensory signals [such as the proprioceptive incongruency between the location of the real hand and the rubber hand in the case of the rubber hand illusion paradigm; (Knoblich, 2006; Makin et al., 2008; Tsakiris, 2010)].

Numerous studies have shown that immersive VR is an especially powerful tool to alternate body perceptions. The rubber hand illusion has, for example, been replicated numerous times in VR –in the so-called “virtual hand illusion”– where congruent (e.g., haptic or tactile) feedback is provided to the real hand of the participant together with visual feedback in VR [i.e., on the hand of the avatar seen by the participant; (Slater, 2008; Perez-Marcos et al., 2009; Sanchez-Vives et al., 2010; Pyasik et al., 2020; Jeong and Kim, 2021; Kanayama et al., 2021)]. Further, the high visuo-motor or visuo-proprioceptive synchrony –i.e., the high spatial and temporal correlation between the performed movement and the visually perceived feedback on the display– in immersive VR has also been shown to induce strong embodiment over the avatar, without the need of additional tactile stimulation (Sanchez-Vives et al., 2010; Carey et al., 2019; Odermatt et al., 2021). Notably, immersive VR, together with additional sensory feedback, can be used to induce embodiment over “unrealistic” avatars (Kilteni et al., 2012b; Preston and Newport, 2012). For example, Kilteni et al. (2012b) induced a “very long arm illusion” by visually elongating a virtual arm and simultaneously providing haptic feedback to the real hand of the participant which was visually reproduced in the VR (namely, the touching of a grass surface).

Importantly, body perceptions impact how people interact with the environment. When we perform actions, it is critical to keep track of, for example, the size and shape of the different body parts (Head and Holmes, 1911; Holmes and Spence, 2004; Maravita and Iriki, 2004). In a series of experiments, Tajadura-Jiménez et al. (2012, 2015b,2016) used real-time auditory feedback to induce illusionary ownership over elongated arms (e.g., by providing sounds that implied to originate from a greater distance when participants tapped their hand on a surface). Crucially, the authors showed that the illusion of having a longer arm also influenced the real arm movements of participants, similarly to what would be expected if the illusionary body characteristics were real (Tajadura-Jiménez et al., 2016). Further, in another study by Kilteni et al. (2013), the authors showed that participants who embodied dark-skinned avatars using immersive VR improved their drumming patterns compared to light-skinned avatars, therefore, not only showing that participants expected people with darker skin color to be better at drumming than people with lighter skin color, but also that they embodied the darker-skinned avatar.

The finding that motor actions are influenced by manipulating the self-perception of the own body using immersive VR may have important applications for neurorehabilitation. The flexibility of the brain regarding embodiment could be exploited to induce the feeling of body ownership over avatars with different body characteristics, modulating underlying motor brain networks and performance and optimizing recovery. For example, embodying a virtual stone arm may increase the physical engagement during training, similarly to lifting an empty bottle that is believed to be full. However, the ability of the brain to adapt motor commands based on a perceived modified reality has not yet been fully explored. Evidence suggests that the embodiment over an artificial limb may go in line with the disembodiment of the own limb (for a review see Golaszewski et al., 2021). Previous neurophysiological studies using non-invasive brain stimulation techniques (transcranial magnetic and direct current stimulation) and electroencephalography (EEG) have evidenced attenuated activity in motor (della Gatta et al., 2016; Fossataro et al., 2018) and somatosensory (Tajadura-Jiménez et al., 2012; Zeller et al., 2015; Hornburger et al., 2019; Isayama et al., 2019; Sakamoto and Ifuku, 2021) brain areas, along with enhanced error tolerance (Raz et al., 2020) during the experience of illusionary body ownership. However, most studies on the neural correlates underlying embodiment investigated illusionary body ownership over a rubber hand (Botvinick and Cohen, 1998; Ehrsson, 2004; Tsakiris and Haggard, 2005; Haans et al., 2008). Importantly, in the rubber hand illusion paradigm, the hand of the participant is not located at the same place as the rubber hand. To overcome this proprioceptive mismatch and to embody the rubber hand, the brain may be forced to disembody the own hand, lowering neural activity in the corresponding brain areas. Further, the experience of illusionary body ownership is commonly associated with congruent multisensory feedback (for example, applied to the participants real hand and a rubber hand), while the control condition (i.e., low body ownership) is associated with incongruent feedback, previously shown to introduce confounding congruency effects (Rao and Kayser, 2017; Odermatt et al., 2021). Yet, immersive VR allows for congruent multisensory feedback with high visuo-proprioceptive congruency –i.e, the motor actions are spatially and temporally highly correlated with the visual feedback perceived through the HMD– and therefore, disembodiment of the own limb may not be necessary, allowing for a more naturalistic embodiment. This is in line with previous work showing that body illusions based on unimodal sensory feedback and without proprioceptive mismatch enhance activity in motor brain networks: visual kinesthetic illusions, in which the illusory feeling of motion of a static body part is induced by mechanically vibrating the tendon muscle of a physically constrained joint, have been associated with an increase in motor cortical excitability (for a review see Dilena et al., 2019). Therefore, in this study, we aimed at “tricking the brain” in a naturalistic fashion using immersive VR and investigate if multisensory feedback modulating the physical properties of an embodied avatar influences motor brain networks and control. To allow for a more naturalistic embodiment, we decided to change the skin material rather than the limb size or shape (e.g., elongated arm), to not introduce a proprioceptive mismatch during the illusion which could be associated with reduced motor activity.

Ten healthy participants were immersed in VR with an HMD, where they saw an avatar from a first-person perspective. We applied multisensory feedback (i.e., auditory, tactile, and visual) to induce a “stone arm illusion,” inspired by the work of the marble hand illusion by Senna et al. (2014). We slowly transformed the surface of the avatar (i.e., the “skin material”) from human to stone. We enforced this visual change by repetitively touching the arm of the participant and the real arm of the avatar with a (virtual) hammer, while progressively replacing the sound of a hammer against skin with the sound of a hammer against stone provided via a speaker. To study changes in motor brain networks associated with the illusion, we applied single-pulse transcranial magnetic stimulation (TMS) over the primary motor cortex. Applying TMS through the scalp over the primary motor cortex elicits action potentials in motor neurons of the brain, which can be captured as motor evoked potentials (MEPs) with electromyographic recordings on the corresponding muscles (Rothwell, 1997; Wolf et al., 2005; Groppa et al., 2012). Amplitude and latency of the MEPs are influenced by the cortical excitability of the motor system and corticospinal tract and can therefore be used as an index of physiological state changes in the primary motor area (Barker et al., 1985; Rothwell et al., 1987a,b; Bestmann, 2012; Bestmann and Krakauer, 2015; Rossini et al., 2015; Schmidt and Brandt, 2021). Further, to investigate if the “stone illusion” affected action execution, participants performed a reaching task visualized in VR with the human and stone avatar, i.e., they had to reach as fast and accurately as possible from a resting hand position on a table to appearing spheres above the table. Finally, we used questionnaires to assess the subjectively reported strength of the embodiment and illusion.

We expected that the immersive, highly congruent multisensory feedback in VR would induce strong body ownership over the avatar across both the human and stone conditions. In addition, we expected higher subjectively rated “stone feeling” in the stone versus human avatar condition, indicating the presence of a “stone arm illusion.” We further hypothesized that the stone arm illusion or the subjectively experienced stone feeling would be associated with enhanced motor cortical excitability, reflecting an adaptation of motor brain processes to the altered body image. Further, we hypothesized that the stone arm illusion or stronger subjectively experienced stone feeling would enforce accelerated movement patterns and/or motor overshooting in the reaching movements, due to an overestimation of the weight of the real arm. Finally, to better understand the nature of the stone arm illusion, we were further interested in exploring the relationship between the stone feeling and embodiment components (as stronger subjectively experienced stone feeling may hamper agency but not body ownership), and between the reaching movements and cortical excitability in the human and stone condition.

## Materials and Methods

### Participants

We recruited 10 healthy participants [five female; age (M ± SD) = 29.4 ± 6.5 years] from the campus of the University of Bern, Switzerland. All participants reported to be right-handed when asked to indicate their dominant hand and to have normal or corrected-to-normal vision. None of them had a psychiatric or neurological clinical history. Participants were naïve to the hypotheses of the experiment. The study was approved by the local ethics committee and all participants gave written informed consent.

### Experimental Setup

#### Material

**FIGURE 1:**
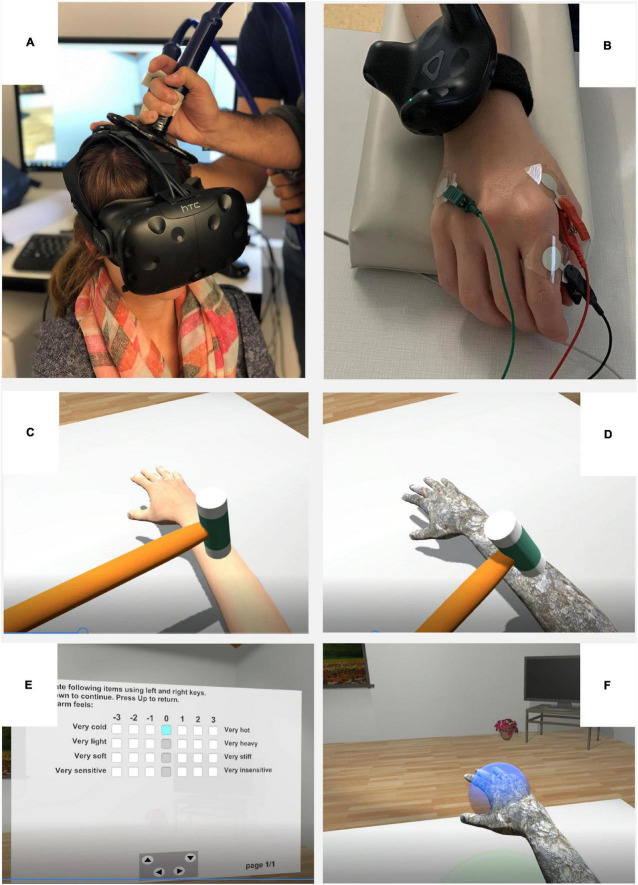
Experimental setup and virtual environment. **(A)** Participant wearing the head-mounted display (HMD) and receiving transcranial magnetic simulation (TMS) over the primary motor cortex. **(B)** Electromyographic recordings in the shape of MEPs elicited by the TMS pulses were obtained from the first dorsal interosseous (FDI) muscle of the right hand of the participant placed on an armrest and with the tracker around wrist and upper arm. **(C)** The first-person perspective point of view of the participant in the VR during the multisensory feedback in the human and **(D)** stone condition. **(E)** The first-person perspective of the participant during the questionnaires, and **(F)** the motor task.

An overview of the experimental setup can be seen in Figure 1. A head-mounted display (HTC Vive, HTC, Taiwan and Valve, United States), two trackers, and one controller (HTC Vive, Taiwan and Valve, United States) were employed in the VR setup. Two trackers were attached on the right upper arm and wrist of the participant with Velcro^®^ straps to record the motion kinematic data and visually animate the avatar in the VE. The controller was operated by the experimenter to animate a virtual hammer. The kinematic data of the trackers were continuously collected at a sampling rate of ∼50 Hz in the Unity game engine and stored for offline analysis (version 2018.3.0f2; Unity Technologies, United States).

A 4-button response box (The Black Box ToolKit Ltd., United Kingdom) placed on a table was used by the participants to answer the questionnaires in VR. A loudspeaker located on the right side of the same table provided the auditory feedback. The data obtained via response box were collected in the Unity game engine and stored for offline analysis.

A Magstim 200 Mono Pulse stimulator (Magstim Ltd., United Kingdom) and a figure-of-eight coil were used to apply TMS pulses through the scalp of the head of the participant over the primary motor area (Figure 1A). A TMS navigation system (Localite GmbH, Germany) was employed for the co-registration of the position and orientation of the coil with the head of the participant. Electromyographic recordings in the shape of MEPs elicited by the TMS pulses were obtained using the Dantec Keypoint G4 Workstation (Natus Medical Incorporated, United States) from the right hand of the participant in a belly-tendon montage by means of Ag/AgCl surface tab electrodes with a diameter of 5 mm (Medtronic Ltd., United Kingdom). The active electrode was placed over the belly of the first dorsal interosseous (FDI) muscle, the reference electrode over the proximal interphalangeal joint of the index finger (tendon), and the ground electrode over the abductor digiti minimi (Figure 1B). The electromyographic raw signal was amplified, recorded with a sampling rate of 48 kHz, and stored for offline analysis using Keypoint. Net Software (version 2.31; Natus Medical Incorporated, United States).

#### Virtual Environment and Avatar

The virtual environment was built in Unity game engine (version 2018.3.0f2; Unity Technologies, United States) and consisted of a virtual living room. A male and a female avatar were designed in MakeHuman (open source software version 1.1.1)^1^. Participants saw the gender-matched avatar from a first-person perspective sitting on a chair in front of a table, i.e., they could see the upper body (arms, shoulder) and parts of the legs of the avatar (Figures 1C,D). The right arm of the avatar was animated using the position of the trackers of HTC Vive placed on the right upper arm and wrist of participants. The left arm of the avatar was rendered to be located under the virtual table (i.e., the left hand of the avatar was neither animated nor visible in VR). A controller operated by the experimenter was employed to animate a virtual hammer.

### Experimental Procedure

The whole experiment was completed in a single session with a total duration of approximately 60–70 min. Participants were seated comfortably at a table with their right hand placed on a soft armrest in a predefined position in front of them, matching the hand of the avatar on the virtual table in VR.

**FIGURE 2:**
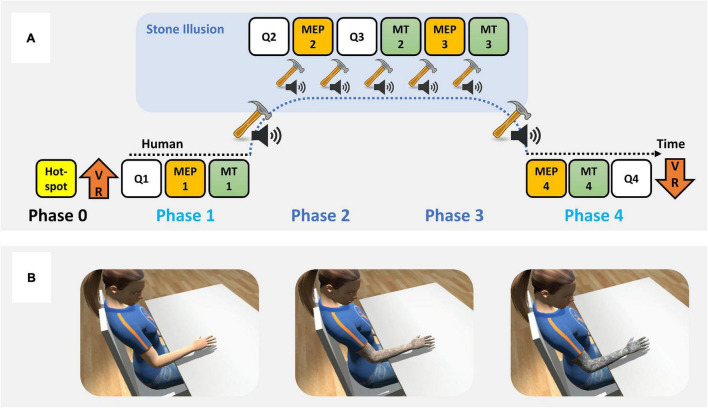
Experimental procedure. **(A)** Experimental protocol, and **(B)** exemplar overview of the virtual environment, including the female version of the avatar with animated human surface (left), mixed surface during the transformation (middle), and stone surface (right).

The experiment consisted of five phases, i.e., a baseline phase (phase 0) and four experimental phases (phases 1–4; Figure 2A). Task instructions were presented outside VR before the start of the experiment. The baseline phase was performed outside VR to assess the motor hotspot for the TMS application on the head of the participant [see section “Motor Hotspot Definition (Phase 0)”]. Then, participants were immersed in VR with their right hand placed on the armrest and the left hand on the response box (to fill in the questionnaires). Before starting the experiment, participants could visually explore the virtual environment. In each experimental phase, participants performed three measurement blocks, i.e., a questionnaire block [see section “Questionnaire Blocks (Phases 1–4)”], an MEP evaluation block [see section “Motor Evoked Potential Evaluation Blocks (Phases 1–4)”], and a motor task block [see section “Motor Task Blocks (Phases 1–4)”], while continuously being immersed in VR. The phases or blocks were manually initiated by the experimenter. Phases 1 and 4 were performed with the avatar animated with human skin while phases 2 and 3 were performed with the avatar animated with a stone surface (Figure 2B).

The first experimental phase performed with a human-skinned avatar (i.e., first human avatar condition) started with the questionnaire block (QT1). Then, participants underwent the first MEP evaluation block (MEP1) and finished with a motor task block (MT1). After phase 1, participants received the multisensory feedback for approximately 50 s during which we induced the “stone arm illusion” [see section “Experimental Conditions (Phases 1–4)”]. After the skin transformation was finished, phases 2 and 3 (i.e., first and second stone arm conditions) started with alternating multisensory feedback and measurement blocks. First, participants answered the questionnaires (QT2), followed by the MEP evaluation (MEP2), another questionnaire (QT3), and a motor task block (MT2). Then, participants received another MEP evaluation block (MEP3), followed by a motor task block (MT3). Between each experimental block in phases 2 and 3, participants received multisensory feedback for 15 s, i.e., they felt/saw a hammer touching their real/the arm of the avatar triggering a stone sound from the speaker. The order of phases 2 and 3 was selected to prioritize the TMS evaluation and questionnaire ratings over the motor task, in case the reaching movements would break the illusion. After phases 2 and 3, we transformed the avatar back to a human skin surface by applying the multisensory feedback for around 50 s [see section “Experimental Conditions (Phases 1–4)”]. Finally, the fourth phase started (i.e., second human avatar condition), where participants first received the MEP evaluation (MEP4), then performed the motor task (MT4) and finished by filling in the questionnaires (QT4) for the last time. Finally, participants were taken out of the immersive VR and debriefed about the study aim.

#### Motor Hotspot Definition (Phase 0)

Before the experimental phases in the VR environment, we determined the location of the “motor hotspot,” i.e., the stimulation site on the head of the participant reliably producing high amplitude TMS-induced MEPs recorded from the FDI muscle of the right hand of the participant. Participants were asked to relax the muscles in arm and hand. Complete muscle relaxation was monitored via audiovisual feedback. Then, single-pulse TMS was applied by a blinded experimenter (i.e., naïve to the experimental conditions) over the primary motor cortex. Stimulation intensity started at 10% and was slowly increased in increments of 2–5%. The region over the skull where the stimulation induced reliable MEPs of the first dorsal interosseous muscle activation across 10 consecutive trials was defined as the “motor hotspot.” Mean stimulation intensity across all participants was 49% (SD = 6.5; range 40–57%). The coil position of the hotspot was marked directly on the scalp to ensure accurate coil repositioning. Since efficiency (i.e., the stimulus intensity required to bring corticospinal neurons to firing threshold) and type (i.e., direct axonal versus indirect trans-synaptic) of TMS stimulation are highly influenced by the orientation of the neural element within the induced electric field (Bonato et al., 2006; Thut et al., 2011), we co-registered the M1 hotspot location on the participants head with the TMS coil using a neuronavigation system (Localite GmbH, Germany). The whole baseline procedure took around 10–15 min.

#### Experimental Conditions (Phases 1–4)

The two experimental conditions represent the embodiment of a human arm/hand avatar and a stone avatar, respectively, which we modulated using congruent multisensory feedback. After the experimental phase 1 (resp., after phase 3), we induced a “stone arm illusion” (resp. “human arm illusion”) by gradually transforming the surface of the avatar (i.e., the “skin material”) from human to stone (resp., from stone to human; Figure 2B). We enforced this visual change by gently and repetitively touching at ∼1 Hz the real forearm of the participant with an HTC Vive controller while touching the forearm of the avatar in the VR with a virtual hammer animated using the position and orientation of the controller. We progressively replaced the sound of a hammer against skin displayed from the loudspeaker on the table with the sound of a hammer against stone (resp., vice versa; see “Supplementary Material” for an exemplar video). The stone hitting sound was generated by recording the sound of a real hammer hitting a real stone. The human skin hitting sound was generated by recording the sound of a real hammer hitting a real arm. Of note, the tactile feedback (i.e., the touch with the controller on the forearm of the participant) did not change across transformation. The transformation lasted for approximately 50 s.

#### Questionnaire Blocks (Phases 1–4)

Participants filled in two questionnaires to assess the subjectively reported embodiment and the perceptual correlates of the stone arm illusion (i.e., stone feeling). Questionnaires were presented in VR to keep them standardized and to facilitate immersion and answered by the participants with the left hand on the response box (Figure 1E).

The stone feeling questionnaire consisted of four items on a 7-point Likert scale, indicating how cold/hot, light/heavy, soft/stiff, and sensitive/insensitive participants rated their right arm (see Table 1). The questionnaire was adapted from the study by Senna et al. (2014). The embodiment questionnaire consisted of eight items adapted from established questionnaires (Longo et al., 2008; Bassolino et al., 2018) that were rated on a 7-point Likert scale from −3 (strongly disagree) to 3 (strongly agree). The three main components of embodiment (body ownership, agency, and location) and disownership were assessed. In addition, control items unrelated to the body illusion were included to validate the specificity of potential illusion effects (Table 2). Participants took around 1–3 min to fill in the questionnaires.

**TABLE 1:** Stone feeling questionnaire.

Item	Dimension	“My right arm feels”								
			−3	−2	−1	0	1	2	3	
I1	Coldness	very cold								very hot
I2	Heaviness	very light								very heavy
I3	Stiffness	very soft								very stiff
I4	Insensitivity	very sensitive								very insensitive

*Adapted from Senna et al. (2014).*

**TABLE 2:** Embodiment questionnaire.

Items	
**Body ownership**	
Q1	It seemed like the virtual arm was my arm
Q2	It seemed like the virtual arm was part of my body
**Location**	
Q3	It seemed like my arm was in the location where the virtual arm was
**Agency**	
Q4	It seemed like I was in control of the virtual arm
**Disownership**	
Q5	It seemed like the experience on my real arm was less vivid than normal
Q6	It seemed like my real arm had disappeared
**Control items**	
Q7	It seemed like I had more than two arms
Q8	It seemed as if my real arm was becoming virtual

*Q1–4, Q6–8 (Longo et al., 2008); Q5 (Bassolino et al., 2018).*

#### Motor Evoked Potential Evaluation Blocks (Phases 1–4)

During the MEP evaluation blocks, participants received single-pulse TMS over the left primary motor cortex, i.e., contralateral to the electromyographic leads at the marked optimal site [i.e., motor hotspot, see section “Motor Hotspot Definition (Phase 0)”] for first dorsal interosseous muscle activation of the right hand. The consistent coil orientation across MEP blocks was verified using a neuronavigation system (Localite GmbH, Germany). A total of 20 ± 2 TMS pulses were applied, and the corresponding MEPs recorded in each block with an inter-pulse interval of approx. 3 s. The total duration of an MEP evaluation block was around 2 min.

#### Motor Task Blocks (Phases 1–4)

Participants were asked to perform a motor task consisting of reaching as fast and accurately as possible with their right arm or hand placed on the armrest located on the table to vertically appearing blue spheres (Figure 1F). The resting initial position was indicated with a green sphere in the virtual environment. After reaching to a blue sphere, participants were asked to bring back their hands to the rest position (green sphere) until a next blue sphere appeared. One block consisted of four trials/blue spheres, i.e., two blue spheres placed 32 cm and two blue spheres placed 36 cm above the table (i.e., 20 and 24 cm above the resting position/armrest, respectively). The two different reaching distances were selected to minimize the possibility of potential movement anticipation strategies of participants. All blue spheres were placed above the initial position of the hand resting on the table (i.e., the vertical projection over the green sphere). The order of the spheres was randomized to minimize anticipation. One motor task block lasted for around 1 min.

### Metrics and Data Processing

#### Stone Feeling

To quantify the subjectively experienced stone feeling, the mean of the coldness (of note, this item was reversed for the analyses, so that positive values reflect coldness), heaviness, stiffness, and insensitivity item ratings for the human and stone condition were calculated for each participant.

#### Embodiment

To quantify the subjectively experienced level of embodiment over the avatar, the mean of the body ownership (Q1–Q2), agency (Q4), location (Q3), disownership (Q5–Q6), and control items (Q7–Q8) were calculated for each participant and condition (i.e., human, stone).

#### Cortical Excitability

Cortical excitability was quantified using the peak-to-peak amplitude from the TMS-induced MEPs (Di Lazzaro and Rothwell, 2014; Schulz et al., 2014; Bestmann and Krakauer, 2015; Rossini et al., 2015; Smith et al., 2019; Ammann et al., 2020). The peak-to-peak MEP amplitude (mV) was calculated as the voltage difference between the maximum positive and maximum negative peak in the electromyographic potential occurring 15–80 ms after TMS pulse onset and averaged across participants and conditions (i.e., human and stone).

#### Kinematic Variables

Due to the uneven sampling rate in Unity (∼50 Hz), data were linearly interpolated every 15 ms (= 66.67 Hz). We calculated the maximum speed (*m/s*), the time to the maximum speed (*s*), the maximum acceleration (*m/s*^2^), and path length (*m*) of the reaching movements. We selected these kinematic variables based on previously used ones in literature to quantify motor performance (Shishov et al., 2017; Basalp et al., 2021). Since we expected that the real sensory feedback during the motor task may break the stone illusion (i.e., due to visuo-motor or visuo-proprioceptive synchrony), we calculated the kinematic variables for both, the first 150 ms after movement onset (a period in which the cerebellum is assumed to not have received updated sensory feedback; Miall et al. (2007); and therefore, reflecting feedforward kinematics associated with movement initiation) and from movement onset until the visual outer border of the sphere was crossed (defined by a collider in Unity). Further, the time (*s*) from movement onset until the visual outer boarder of the sphere was reached (defined by a collider in Unity) was computed. Finally, motor overshooting (*m*) was quantified by calculating the highest point reached by the center of the hand in the upward movement after movement onset minus the height of the center of the blue sphere. Movement onset was defined as the time point when 2% of the maximum velocity after the presentation of the sphere was reached. Each kinematic variable was averaged per participant and condition (i.e., human, stone).

### Data Analysis

The data of both human arm (phases 1 and 4) and stone arm (phases 2 and 3) conditions were averaged for each participant to account for the time factor, which may be associated with intra-subject habituation or fatigue effects across experimental phases.

To investigate whether the stone feeling and the embodiment questionnaires, MEP amplitudes, and the kinematic variables differentiated between the human and stone condition, parametric (paired *t*-tests) and non-parametric (Wilcoxon Signed-Rank tests) pairwise comparisons were performed when applicable.

Further, Pearson product-moment or Spearman’s rank correlation analyses (depending on the statistical distribution of the datasets) were conducted to study the relationship between (1) stone feeling items and MEP amplitudes, (2) stone feeling items and kinematic variables, (3) stone feeling items and embodiment components, and (4) kinematic variables and MEP amplitudes. Correlation analyses were performed separately for the human and stone condition.

Assumptions for parametric testing were checked using normality tests (Kolmogorov–Smirnov, *p* > 0.05). Outlier trials (more than ± 2.5 SDs from the mean of the participant) were excluded from the analyses. All *p* values were corrected for multiple hypothesis testing using Tukey–Kramer and Bonferroni–Holm, respectively (between conditions comparisons) and the Benjamini-Hochberg false discovery rate (correlation analyses). Statistical analyses were performed with *R* v. 4.1.1 and the significance threshold was set at α < 0.05. If not otherwise stated, two-sided hypothesis testing was applied (and we indicate one-sided testing in the case there was a clear directed *a priori* hypothesis).

## Results

A summary of the results with the statistics is represented in Table 3.

**TABLE 3:** Descriptive statistics and results of the pairwise comparisons.

Variables	Human condition	Stone condition	t/z	*p* value
**Stone illusion**				
Coldness	0.0 (1.08)	0.75 (1.12)	2.5 (t)	0.02*
Heaviness	0 (−1 to 0)	1 (0–1)	2.10 (z)	0.036*
Stiffness	−0.1 (1.12)	0.75 (1.37)	2.99 (t)	0.016*
Insensitivity	0 (−0.25 to 0)	0 (0–1)	−2.44 (z)	0.02*
**Embodiment**				
Body ownership	4.78 (1.16)	4.35 (1.42)	−1.56 (t)	0.22
Agency	6 (5–6.25)	5.5 (5–7)	−1.26 (z)	0.26
Location	5.9 (0.94)	5.4 (1.53)	−1.81 (t)	0.22
Disembodiment	3.48 (1.78)	3.85 (1.16)	1.58 (t)	0.22
Control	3.38 (0.72)	3.2 (0.8)	−0.82 (t)	0.42
**Cortical excitability**				
MEP Amplitude (*mV*)	0.95 (0.81–1.82)	1.03 (0.83–1.87)	−0.04 (z)	0.49
**Feedforward kinematics (movement initiation)**				
Max speed *(m/s)*	1.14 (0.44)	1.3 (0.44)	−1.53 (t)	0.14
Time to max speed (*s*)	0.12 (0.11–0.14)	0.14 (0.13–0.14)	−2.28 (z)	0.048*
Max acceleration (*m/s*^2^)	12.93 (6.83)	14.31 (7.11)	−1.18 (t)	0.16
Path length (*m*)	0.1 (0.04)	0.11 (0.04)	−0.76 (t)	0.23
**Movement until sphere**				
Max speed (*m/s*)	1.11 (0.98–1.78)	1.17 (1–1.97)	−2.82 (z)	0.09
Time to max speed (*s*)	0.15 (0.14–0.19)	0.15 (0.14–0.17)	−0.20 (z)	0.84
Max acceleration (*m/s*^2^)	11.25 (8.2–20.34)	11.55 (8.55–21.38)	−1.01 (z)	0.62
Path length (*m*)	0.19 (0.05)	0.2 (0.07)	−0.46 (t)	0.84
**Motor overshooting**				
Height above sphere (*m*)	0.05 (0.01)	0.05 (0.02)	0.40 (t)	0.48

*Mean (standard deviation) or median (25% quantile–75% quantile) range are reported.*

*Indicates significance at the 0.05 level.

### Between Conditions Differences

#### Stone Illusion

**FIGURE 3:**
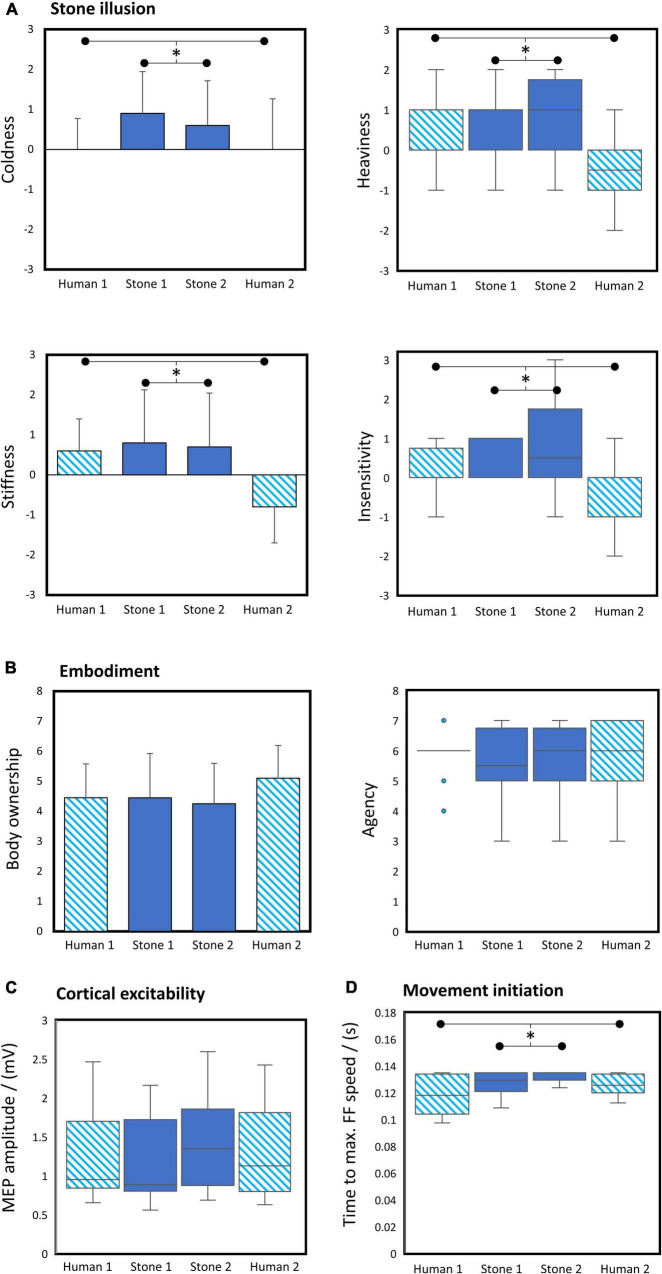
Between conditions differences. **(A)** Rated stone feeling, **(B)** subjectively experienced embodiment, **(C)** cortical excitability assessed via motor evoked potential (MEP) amplitudes, and **(D)** time to the maximum speed in the feedforward kinematics reflecting movement initiation across phases. H1, first human condition; S1, first stone condition; S2, second stone condition; H2, second human condition. Bar plots: Error bars represent standard deviation. Boxplots: Whiskers show the data ranging 1.5 times inter-quartile range above the upper or below lower quartiles, boxed horizontal solid lines show the median and box vertical boundaries show the inter-quartile range. *p < 0.05 for pairwise comparisons between human (mean H1 + H2) and stone (mean S1 + S2) condition.

Pairwise comparisons showed significant (one-sided) stone illusion effects. Subjects rated their right arm to be colder, heavier, stiffer, and more insensitive in the stone versus human condition (Figure 3A).

#### Embodiment

Pairwise comparisons did not show significant differences in the subjectively reported embodiment components (i.e., body ownership, agency, and location), disembodiment, and control items between the human and stone condition (Figure 3B).

#### Cortical Excitability

The pairwise comparison did not reveal a significant (one-sided) modulation of the MEP amplitude between the human and stone condition (Figure 3C).

#### Kinematic Variables

We found a significant (one-sided) effect of the illusion on the time until the maximum speed in the feedforward kinematics, which was higher in the stone than in the human condition (Figure 3D). None of the other kinematic variables showed significant differences between the human versus stone avatar condition in the pairwise comparisons.

### Correlation Analyses

#### Stone Feeling and Motor Evoked Potentials

**FIGURE 4:**
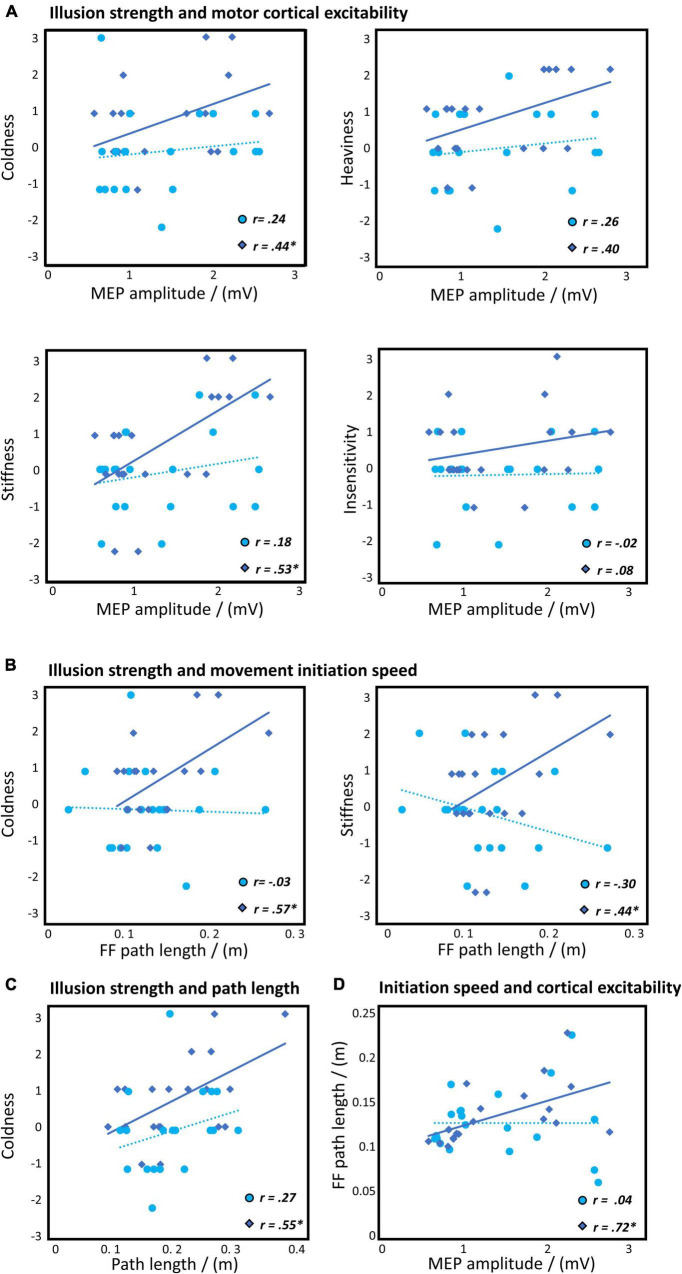
Results of the correlation analyses for the human (in lighter blue/circles) and stone (in darker blue/diamonds) condition. **(A)** Stone illusion strength and MEP amplitude reflecting cortical excitability. **(B)** Stone illusion strength and feedforward (FF) path length reflecting the average speed in the movement initiation. **(C)** Stone illusion strength and path length for the movement until the sphere. **(D)** Average speed in the feedforward kinematics and cortical excitability. Of note, due to few very similar values across human and stone conditions, the number of individually visible plots (i.e., circles and diamonds) may be lower than the number of measurement points (i.e., 20). *p < 0.05.

Correlation analyses revealed a significant relationship between the subjectively reported stone feeling with the MEP amplitude in the stone but not the human condition (Figure 4A). The stronger the rated coldness [*r*_s_(18) = 0.44, *p* (one-sided) = 0.03] and stiffness [*r*_s_(18) = 0.53, *p* (one-sided) = 0.02], the higher was the cortical excitability. We further found a trend for an association between the rated heaviness with the MEP amplitude [*r*_s_(18) = 0.40, *p* (one-sided) = 0.08]. The MEP amplitudes were not associated with the insensitivity item of the stone feeling [*r*_s_(18) = 0.08, *p* (one-sided) = 0.5].

#### Stone Feeling and Embodiment

A significant negative correlation between the stone feeling and agency (but not body ownership) was observed in the human and stone conditions. In the stone condition, coldness ratings were associated with reduced agency [*r*_s_(18) = −0.56, *p* = 0.02]. In the human condition, insensitivity was associated with reduced agency [*r*_s_(18) = −0.55, *p* = 0.02], while there was a trend for the heaviness [*r*_s_(18) = −0.41, *p* = 0.08].

#### Stone Feeling and Kinematic Variables

We also found significant positive correlations between the stone feeling items with kinematic variables in the stone but not human condition. The higher the rated coldness [*r*_p_(18) = 0.57, *p* (one-sided) = 0.02] and stiffness [*r*_p_(18) = 0.44, *p* (one-sided) = 0.03] of the arm of the subject, the longer were the performed paths within the 150 ms after movement onset in the stone condition (Figure 4B). Further, the coldness item was associated with longer paths in the movements until the sphere in the stone condition [*r*_p_(18) = 0.55, *p* = 0.04; Figure 4C].

#### Kinematic Variables and Motor Evoked Potentials

Finally, we found a significant correlation between the feedforward kinematics (i.e., within the 150 ms after movement onset) and MEP amplitudes in the stone but not the human condition. The higher the cortical excitability, the longer were the performed paths [*r*_s_(18) = 0.72, *p* = 0.03; Figure 4D]. For the movements until the sphere, we found significant associations between the kinematics and the MEP amplitude for the stone condition. More precisely, higher cortical excitability was associated with longer paths [*r*_s_(18) = 0.51, *p* = 0.03] and higher maximum acceleration [*r*_s_(18) = 0.48, *p* = 0.03]. Further, we found a trend for an association between the motor overshooting and the MEP amplitudes in the stone condition [*r*_s_(18) = 0.43, *p* = 0.06].

No further correlation analyses reached significance. A summary of the correlation analyses is represented in Table 4.

**TABLE 4:** Significant correlations (*p* < 0.05) between measures for human (H) and stone (S) condition.

Variables		Cortical excitability	Stone feeling			
		MEP amplitude (*mV*)	Coldness	Heaviness	Stiffness	Insensitivity
Cortical excitability	MEP amplitude (*mV*)	−	S+		S+	
Embodiment	Body ownership	−				
	Agency	−	S−			H−
Feedforward kinematics (movement initiation)	Max. speed (*m/s*)					
	Time to max. speed (*s*)					
	Max. acceleration (*m/s*^2^)					
	Path length (*m*)	S+	S+		S+	
Movement until sphere	Max. speed (*m/s*)					
	Time to max. speed (*s*)					
	Max. acceleration (*m/s*^2^)	S+				
	Path length (*m*)	S+	S+			
Overshooting	Height above sphere (*m*)					

*The plus and minus signs indicate if the correlation is positive or negative.*

## Discussion

We “tricked the brain” using immersive VR to investigate if multisensory feedback modulating the physical properties of an embodied avatar influences motor brain networks and control. Ten healthy participants were immersed in a virtual environment using an HMD, where they saw an avatar from first-person perspective. We slowly transformed the visual appearance of the human-skinned avatar to an avatar with a stone surface. To enforce the “stone arm illusion,” we simultaneously applied auditory and tactile feedback during the visual transformation, i.e., participants saw and felt a (virtual) hammer touching their real arm or the arm of the avatar, triggering a progressively changing human to stone hitting sound from a loudspeaker. Participants filled in questionnaires to report their level of embodiment and experienced stone feeling, had single-pulse TMS applied over the primary motor cortex, and performed an arm reaching task to study how the “stone arm illusion” affected motor cortical excitability and action execution.

### The Strength of Subjectively Experienced “Stone Arm Illusion” Is Associated With Enhanced Motor Cortical Excitability

In line with our expectation, our participants indeed experienced the “stone arm illusion.” They rated their arm as colder, heavier, stiffer, and more insensitive when we enforced illusionary ownership over a stone versus human avatar using multisensory feedback in immersive VR. The adaptation of the participants to the stone illusion is further visible in the aftereffects found after the transformation back to the human avatar. Participants rated their own arm as less heavy, stiff, and insensitive after the illusion compared with the baseline (i.e., first human block). The stone illusion is a result of both the relatively enhanced stone feeling during the immersion with the stone compared with the human avatar and the relatively lowered stone ratings below baseline after the transformation back to the human avatar. Importantly, the stone illusion did not impact the experienced level of embodiment. Participants reported high body ownership and agency over the avatar, independently of the condition. Our results are in line with a vast amount of research that used multisensory feedback with (Slater, 2008; Perez-Marcos et al., 2009; Kilteni et al., 2012b,^2016^; Pyasik et al., 2020; Tambone et al., 2021) and without immersive VR (Botvinick and Cohen, 1998; Ehrsson, 2004; Petkova and Ehrsson, 2008; van der Hoort et al., 2011; Burin et al., 2017) to induce various body illusions. These findings established the view that body perceptions are continuously updated in the brain in response to sensory signals related to the body (Blanke, 2012; Tsakiris, 2017). A certain flexibility of the brain regarding body perception is indeed crucial to maintain a stable body image despite that body characteristics constantly change in response to external influences such as light, temperature, and posture.

We further showed that the strength of the reported stone feeling was associated with enhanced cortical excitability, i.e., with an increased amplitude of the TMS-induced motor evoked potentials in the stone but not human avatar condition. More precisely, the subjectively rated coldness and stiffness of the own arms of the participants in the stone condition were associated with enhanced motor excitability, while we found a tendency for the rated heaviness to be associated with the MEP amplitudes. This finding may indicate that participants physically mirrored the embodied body characteristics of the stone avatar. Participants may have enhanced the muscle tension or activity in their own arm, (unconsciously) mimicking the stiffness of the stone avatar with increasing illusion strength. Cortical excitability is generally thought to reflect the responsiveness of the brain to exogenous and/or endogenous signals (Rosanova et al., 2011). In the case of the primary motor cortex, excitability is linked with a decreased motor threshold and modulated, for example, during action preparation and/or execution (Starr et al., 1988; Bestmann, 2012; Bestmann and Krakauer, 2015). In line with our conclusion, previous studies have shown that muscle contractions enhance primary motor cortical excitability (Arányi et al., 1998; Yahagi et al., 2003; Perez and Cohen, 2008; Perez et al., 2012). However, since our experimental setup was already crowded, we did not add electromyographic recordings during the experiment to objectivate our conclusion on the enhanced muscle tone. Alternatively, the embodied “stone feeling” may have increased the perceived difficulty to control the arm, as task difficulty has previously also been shown to enhance motor cortical excitability (Pearce and Kidgell, 2009; Watanabe et al., 2018).

Importantly, the subjectively experienced illusion strength was only correlated with the MEP amplitude in the stone but not the human avatar condition. The stone avatar condition was further temporally embedded between the human avatar condition, minimizing the possibility that the neurophysiological effects were impacted by confounding factors related to the duration of the experiment, such as room temperature or fatigue (which could be equally expected for the human and stone condition). Since the average cortical excitability did not differentiate between human and stone condition (i.e., in the between conditions analyses), our findings suggests that the MEP amplitudes are crucially modulated during the presence of the stone illusion depending on the subjectively experienced illusion strength.

In line with this conclusion, we found a negative association between subjectively reported stone feeling, namely, the rated coldness, with reported agency, indicating that participants were feeling less in control over their arm, the stronger they experienced the stone illusion. Importantly, the “stone feeling” did not affect the experienced level of body ownership over the avatar. The correlation between the reported stone feeling with the reported embodiment was only present for agency, but not for body ownership and location – the two other embodiment components (Kilteni et al., 2012a). Therefore, the strength of the stone illusion impacted how well participants think they can control their arm, but they kept experiencing the virtual stone arm as their own arm. However, analyses showed a similar pattern for the human avatar condition. Agency (but not body ownership) was also negatively associated with the reported stone feeling in the human avatar condition. Therefore, carry-over effects may have influenced the results, i.e., stone feeling may have persisted in the human condition, hampering the feeling of agency when immersed with the human avatar. Alternatively, other inter-subject variables, such as fatigue or body temperature, which could have also been captured with the questionnaire, may contribute to the reduced reported agency.

To the best of our knowledge, we are the first to show modulated motor brain processing associated with altered body perceptions using immersive VR. Our findings on neurophysiological effects extend previous studies reporting, for example, affective (Tajadura-Jiménez et al., 2015a), (social) cognitive (D’Angelo et al., 2019; Burin and Kawashima, 2021; Clausen et al., 2021; Tambone et al., 2021), and motor (Kilteni et al., 2013; Tajadura-Jiménez et al., 2015a,^2016^) effects of body illusions.

Interestingly, the found enhancement in excitability associated with the strength of the illusionary self-perception suggests a more “complete” body illusion using immersive VR compared with classical paradigms, notably, the rubber hand illusion. Non-invasive brain stimulation studies showed that the activity in motor (della Gatta et al., 2016; Fossataro et al., 2018) and somatosensory (Zeller et al., 2015; Hornburger et al., 2019; Isayama et al., 2019) brain areas was attenuated during the experience of illusionary ownership over a rubber hand (i.e., during the synchronous but not asynchronous multisensory stimulation). These findings have previously been discussed as an indication for the disembodiment of the real hand necessary to embody a rubber hand (for a review see Golaszewski et al., 2021). The use of immersive VR with highly congruent visuo-motor and proprioceptive feedback –compared with the rubber hand illusion, no proprioceptive mismatch is present– may allow to induce highly realistic illusions, without the necessity for the user/brain to disembody the own limbs. As a consequence, the highly realistic visuo-proprioceptive synchrony experienced in immersive VR illusions may influence brain networks similarly as could be expected from modifying real body characteristics (Tajadura-Jiménez et al., 2016).

Together, immersive VR may be an especially powerful tool to realistically modify the perception of the bodily self and influence associated brain networks. Our results show that participants embodied the stone avatar in immersive VR and that the reported illusion strength was associated with the motor cortical excitability, indicative of a physical mirroring of the embodied body characteristics of the stone arm.

### The “Stone Arm Illusion” Influences Movement Initiation in a Reaching Task

We further predicted that the modulated body perception associated with the stone arm illusion using immersive VR would impact motor actions, as body characteristics such as shape and weight critically influence interactions with the environment (Head and Holmes, 1911; Maravita and Iriki, 2004). Our participants performed a simple goal-oriented motor task visualized in the virtual environment, i.e., they had to reach as fast and accurately as possible with their hand from a resting position to vertically appearing spheres. Since we expected that the reaching movement may break the illusion, in addition to the whole movement until the sphere, we analyzed feedforward kinematics within 150 ms after movement onset, in which the cerebellum would not have received updated sensory (e.g., proprioceptive) feedback (Miall et al., 2007).

We found that participants in the stone condition were marginally slower in reaching the maximum speed within the first 150 ms of the movement than when they were in the human condition. Since the maximum speed/path length in the feedforward movement did not differ across conditions, this result indicates an, on average, slightly slower movement initiation when the motor task was performed with the stone versus human avatar. This finding is contrary to what we expected. We predicted that, if the stone illusion worked, participants would overestimate the weight of their real arm, as reflected in enhanced acceleration patterns and motor overshooting, similar to lifting an empty bottle of water that is expected to be full.

However, our correlation analyses suggest that the movement initiation may critically depend on the subjectively experienced illusion strength. The reported stone feeling, namely, the experienced coldness and stiffness, was associated with longer paths within the first 150 ms after movement onset, indicating on average faster movements (average speed defined as the path length over the 150 ms time window). Therefore, participants with stronger illusion effects may have not only stronger physically mirrored (see section “The Strength of Subjectively Experienced ‘Stone Arm Illusion’ Is Associated With Enhanced Motor Cortical Excitability”) but also compensated for the embodied body characteristics of the stone avatar. Stronger experienced illusions might have led participants to put more physical engagement into the task to compensate for the additional (illusionary) weight of the stone arm, resulting in faster feedforward movements or initiations than participants with weaker experienced illusions. In contrast, weak illusion effects during the stone avatar condition could have hampered movement initiation. Participants with relatively weak illusion effects likely experienced an enhanced sensory mismatch in the stone condition than participants with stronger illusion effects. Incongruency of information in virtual environments has previously shown to hamper reaction times and motor performance, independently of the experienced body ownership over the avatar (Odermatt et al., 2021). The facilitated versus hampered feedforward movement depending on the illusion strength could explain why, across the whole group, marginal overall slowed movement initiations were found in the stone versus human avatar condition. Apart from this marginal effect on the movement initiation, the kinematic variables were not influenced by the human versus stone conditions when considering the whole group, suggesting that the physical compensation reflected in the movement initiation may be critically modulated by the subjectively experienced illusion strength.

Our conclusion on a physical compensation of the embodied stone characteristics in the motor task may be further supported by the association between the cortical excitability with faster movement initiation in the stone, but not human condition. The higher the cortical excitability associated with the subjectively reported stiffness and coldness, the faster were the reaching movements 150 ms after movement onset on average (namely, reflected in longer performed paths). It is possible that the previously discussed potential physical mirroring and/or compensation of the embodied body characteristics of the stone avatar “chronically” activated and increased the cortical excitability in motor brain areas, boosting movement initiation. Previous research has shown that the motor cortical excitability is correlated with the force (Baud-Bovy et al., 2008; Perez and Cohen, 2009; Barthélemy et al., 2012) and speed of performed movements (Uehara et al., 2011). However, it needs to be pointed out that the assessment of the cortical excitability was temporally separated from the motor task. Even though literature points toward short-term influences of motor actions on cortical excitability within the range of milliseconds to seconds (Chen et al., 1998; Chen and Hallett, 1999), the exact time course of corticospinal excitability is yet poorly understood.

Our results suggest stronger illusion effects on feedforward kinematics than on the “whole” movement, contrasting previous studies reporting relatively long-lasting motor effects. However, in these studies, the motor task was part of the multisensory feedback to induce the illusion. Tajadura-Jiménez et al. (2012, 2015b,2016), for example, instructed blindfolded participants to tap with their hand on a surface, eliciting sound implying a longer arm (e.g., the provision of lighter sound to simulate increased distance). The authors showed that the illusion of having a longer arm slowed and prolonged the real arm movements of participants. The same authors also found alternated gait patterns when participants had the illusion of owning a lighter versus heavier body modulated with different sounds provided with each footstep (Tajadura-Jiménez et al., 2015a). In both studies, the movements were directly coupled with the real-time auditory feedback modulating the body characteristics. Kilteni et al. (2013), in one of the few studies that used immersive VR to create illusions, showed that participants who embodied dark-skinned avatars exhibited more variable and faster drumming movements compared with embodied light-skinned avatars. Even though, here, no additional sensory enforcement was induced during the motor task, participants were continuously performing the movements. Conversely, in our experiment, the reaching movements were performed between resting periods without the audio-tactile feedback used to enforce the stone arm illusion. Therefore, the (sudden) proprioceptive feedback linked with the movement in the motor task may have more likely disrupted or lowered the illusion effects compared with the setups used in previous studies.

Together, we replicate and extend previous findings with our “stone arm illusion.” We show that a modulated self-perception using multisensory feedback in immersive VR impacts motor control, and may crucially depend on the subjectively experienced illusion strength. Participants with higher reported illusion strength performed, on average, faster reaching movements, indicating that they may have physically compensated for the embodied body characteristics of the stone avatar. In contrast, the incongruent multisensory information associated with weaker experienced illusion effects may have hampered movement initiation.

### Clinical Implications

Our finding that motor brain activity can be influenced based on a perceived modified reality may have important applications for neurorehabilitation. The use of immersive virtual training environments may help to subtly “trick the brain” and change the self-perception of neurologic patients, enhancing the engagement of motor brain networks during motor training. Besides, future studies may investigate to what extent motor brain networks can be activated via the mere immersion into VR environments, without actual motor execution, especially in neurologic patients.

Further, the embodiment of avatars with different body characteristics may offer a playful possibility to implicitly increase the (physical) engagement during training, optimizing motor recovery. However, our results show that the effects of body illusions highly depend on the subjectively experienced illusion strength, and do not necessarily follow from the experimental manipulation of multisensory information. Our findings may be of special relevance for clinical settings considering previous work suggesting that the strength of body illusions in neurologic patients depends on their motor impairment. Using the rubber hand illusion paradigm, Burin et al. (2015) showed that hemiplegic patients reported stronger illusion effects for their impaired hand and weaker effects for the unimpaired hand than healthy controls. The chronic absence of movements may enhance the flexibility of the brain with regard to body ownership for the paralyzed limb, while the healthy limb may be more strictly embodied (Burin et al., 2015). Future studies are needed to investigate how to enforce and optimize body illusions and their behavioral and neural benefits in patients.

### Study Limitations

Similar to previous studies, we did not find consistent illusion effects across different kinematic parameters (Tajadura-Jiménez et al., 2016). As previously highlighted by Tajadura-Jiménez et al. (2016), the small sample size limits the findings and conclusions of the present study. A further reason for the lack of stronger effects found in the kinematic variables may be the experimental setup. The soft armrest on which participants placed their arm during the experiment was slightly sticky and, therefore, noticeable for the participants during the motor task. This may have reduced the immersion in VR, enhancing the attention on the sensory feedback associated with the movement and mitigating illusion effects. In addition, the motor task consisted of only four trials per block (i.e., eight trials per condition) to lower the risk of the performed movements to break the illusion, therefore, conclusions drawn from the movement performance should be treated with caution.

Further, the questionnaire used to assess the stone feeling may have assessed confounds such as inter-subject variability in physical or mental fatigue, motivation, or perceived body temperature. This could also explain why not all stone feeling items showed consistent effects with the experimental manipulation. Future studies may implement additional questionnaires or sensors (e.g., temperature, skin conductance) to objectivate or control for inter-subject confounds.

Moreover, even though we aimed at balancing our two conditions as much as possible to control for intra-subject confounds, for example, related to the duration of the experiment, some differences remained. Participants experienced more audio-tactile feedback during the stone than human condition, to enforce the stone arm illusion between the measurement blocks. The tactile feedback may have increased the awareness of the own arm, in turn, influencing motor brain networks. For example, attention has shown to modulate motor cortical excitability (Conte et al., 2007). Further, the third block was the only block where the MEP evaluation was performed directly after the motor task, and carry-over effects could have impacted our cortical excitability results. However, analysis excluding the third block did not significantly change our findings. This is consistent with findings showing short-lasting effects of movements on cortical excitability (Chen et al., 1998; Chen and Hallett, 1999). In addition, learning effects may confound our results despite our balanced design. Indeed, learning effects were present in most kinematic performance variables. For example, participants showed more accurate (as reflected in shorter path lengths in the movements until the sphere) and faster reaching movements at the end compared with the beginning of the experiment. However, learning may have not occurred linearly across blocks, and therefore, differently affected the performance in the human condition (consisting of the first and last experimental blocks) and the stone condition (consisting of the two embedded experimental blocks). Averaging the blocks of each condition might have, therefore, not fully accounted for learning effects.

Finally, correlation analyses do not reveal the directionality of an association. For example, it is possible that participants with enhanced cortical excitability were more “prone” to experience body illusions and/or to perform faster movements. Similarly, better performance in VR may have enforced the embodiment of the stone avatar (Grechuta et al., 2017, 2019). Future studies are needed to disentangle the directionality and causality of the relationship between VR illusions effects and behavior or neural correlates.

## Conclusion

The goal of this study was to “trick the brain” using immersive VR and to investigate how modulated physical properties of an embodied avatar influence motor brain networks and action execution. Our results show that participants indeed experienced the “stone arm illusion.” The reported illusion strength was associated with enhanced motor cortical excitability and faster movements, indicating that participants may have physically mirrored and compensated for the embodied body characteristics of the stone avatar. Together, alternating the perception of the own body and associated motor brain networks in a subtle way using immersive VR may have important applications for neurorehabilitation and boost the motor recovery of neurologic patients.

## Data Availability Statement

The dataset presented in this study can be found online in the following repository: doi: 10.5281/zenodo.5522866.

## Ethics Statement

The studies involving human participants were reviewed and approved by the Ethics Committee of the Canton of Bern (KEK). The patients/participants provided their written informed consent to participate in this study.

## Author Contributions

KB, JP-A, and LM-C designed the study. JP-A set up the experiment. KB, JP-A, and DC tested all subjects. KB and JP-A analyzed the data. ÖÖ contributed to the analysis of kinematic data. KB and LM-C wrote the manuscript. All authors edited and revised the manuscript and approved the submitted version.

## Conflict of Interest

The authors declare that the research was conducted in the absence of any commercial or financial relationships that could be construed as a potential conflict of interest.

## Publisher’s Note

All claims expressed in this article are solely those of the authors and do not necessarily represent those of their affiliated organizations, or those of the publisher, the editors and the reviewers. Any product that may be evaluated in this article, or claim that may be made by its manufacturer, is not guaranteed or endorsed by the publisher.

## Footnotes

1
http://www.makehumancommunity.org
